# A method for estimating neighborhood characterization in studies of the association with availability of sit-down restaurants and supermarkets

**DOI:** 10.1186/s12942-020-00257-7

**Published:** 2021-03-25

**Authors:** Ke Peng, Daniel A. Rodriguez, Jana A. Hirsch, Penny Gordon-Larsen

**Affiliations:** 1 Department of Urban Planning, School of Architecture, Hunan University, Changsha, Hunan China; 2 Department of City and Regional Planning, University of North Carolina at Chapel Hill, Chapel Hill, NC USA; 3 Department of City and Regional Planning and Institute of Transportation Studies, University of California, Berkeley, Berkeley, CA USA; 4 Urban Health Collaborative, Department of Epidemiology and Biostatistics, Dornsife School of Public Health, Drexel University, Philadelphia, PA USA; 5 Department of Nutrition, Gillings School of Global Public Health, University of North Carolina at Chapel Hill, Chapel Hill, NC USA; 6 Carolina Population Center, University of North Carolina at Chapel Hill, Chapel Hill, NC USA

**Keywords:** Built environment, Sociodemographic, Food stores, Urbanization

## Abstract

**Background:**

Although neighborhood-level access to food differs by sociodemographic factors, a majority of research on neighborhoods and food access has used a single construct of neighborhood context, such as income or race. Therefore, the many interrelated built environment and sociodemographic characteristics of neighborhoods obscure relationships between neighborhood factors and food access.

**Methods:**

The objective of this study was to account for the many interrelated characteristics of food-related neighborhood environments and examine the association between neighborhood type and relative availability of sit-down restaurants and supermarkets. Using cluster analyses with multiple measures of neighborhood characteristics (e.g., population density, mix of land use, and sociodemographic factors) we identified six neighborhood types in 1993 in the Twin Cities Region, Minnesota. We then used mixed effects regression models to estimate differences in the relative availability of sit-down restaurants and supermarkets in 1993, 2001, and 2011 across the six neighborhood types.

**Results:**

We defined six types of neighborhoods that existed in 1993, namely, urban core, inner city, urban, aging suburb, high-income suburb, and suburban edge. Between 1993 and 2011, inner city neighborhoods experienced a greater increase in the percent of sit-down restaurants compared with urban core, urban, and aging suburbs. Differences in the percent of sit-down restaurants between inner city and aging suburbs, high-income suburbs and suburban edge neighborhoods increased between 1993 and 2011. Similarly, aging suburb neighborhoods had a greater percent of supermarkets compared with urban and high-income suburb neighborhoods in 2001 and 2011, but not in 1993, suggesting a more varied distribution of food stores across neighborhoods over time. Thus, the classification of neighborhood type based on sociodemographic and built environment characteristics resulted in a complex and increasingly varied distribution of restaurants and food stores.

**Conclusions:**

The temporal increase in the relative availability of sit-down restaurants in inner cities after accounting for all restaurants might be partly related to a higher proportion of residents who eat-away-from-home, which is associated with higher calorie and fat intake.

## Introduction

Previous studies on access to healthy food have generally characterized neighborhoods by sociodemographic attributes of neighborhood context [1, 2], such as income or race. Although low-income and minority-dominant neighborhoods generally have poor access to healthy food [3], findings on this subject are inconclusive in a U.S. context. For example, some investigators have observed that, compared with moderate and high-income neighborhoods, low-income neighborhoods tend to have greater availability of fast-food restaurants [4, 5], whereas other investigators have not observed a higher prevalence of fast-food restaurants in low-income neighborhoods [6, 7]. In accord with the definition of Caspi et al. [8], herein we refer to *availability* as the presence of certain types of restaurants or food stores in the neighborhood. This definition does not include the degree of ease of getting to food outlets, the food prices, or a person’s attitude about whether the supply of products meets their standards. In addition, there is a novel trend of combining a variety of sociodemographic or socio-economic factors as composite indices [2, 9, 10] to characterize neighborhoods and relate such indices to food availability. However, a common weakness of previous studies has been that they failed to adequately address built environment factors that relate to the type and distribution of food outlets, such as population density and land use pattern. For example, purveyors of some types of restaurants and food stores may have chosen to locate in lower income neighborhoods because residential densities were sufficiently high to maintain demand [11]. A few recent studies [9, 12] in Spain and western Australia (Perth) have combined built environment with sociodemographic factors to characterize neighborhoods. Such studies classified the neighborhoods by, for example, predefined level of development stage first and then sub-classified newly-developed neighborhood by income level (high, medium, low). This scheme raises an issue as to whether there is a clear line between, for example, newly-developed neighborhoods and old neighborhoods [13].

In fact, we know little about neighborhoods defined by a multidimensional categorization that acknowledges the patterning of neighborhoods across many interrelated built environment and sociodemographic characteristics [14]. Because neither aggregate indices of sociodemographic factors nor specific aspects of the built environment appear in isolation in neighborhoods [15], we used a grouping technique, namely cluster analysis, to classify neighborhood types by a combination of several domains. Although cluster analysis has been widely used as a typical approach to classify data into groups, it has less frequently been used to characterize neighborhoods based on multiple interrelated sociodemographic and built environmental variables. Cluster analysis can account for a broad set of neighborhood facility variables to fully capture multiple neighborhood dimensions. Thus, cluster analysis can be used to disentangle the mixed results derived from different neighborhood types. This measurement strategy identifies groups of neighborhoods with shared characteristics (such as population density, mix of land use, and sociodemographic factors) that may be associated with restaurant or food store location within the group of neighborhoods. Thus, similar to previous studies [14–16], we used cluster analysis as a strategy to define neighborhood types and document their patterns of restaurants and food stores.

We aimed to examine the association between baseline neighborhood characterization and change in neighborhood food availability while accounting for the effects of many interrelated aspects of neighborhoods associated with food access. Using the baseline-change method of analysis, we examined the distribution of types of restaurants and food stores within each type of neighborhood to determine whether a particular neighborhood type had relatively greater access to a specific type of restaurant or food store compared with other neighborhood types over three observational years. We make two major contributions to the food access literature. First, we acknowledge that neighborhoods are patterned by interrelated features; thus, we assemble neighborhoods into homogenous groups instead of relying on a couple of pre-specified factors and cut-off levels (e.g., high, medium, low) to find the homogenous neighborhoods. Second, using this characterization, we documented and compared the patterns of restaurants and food stores over a span of 18 calendar years, which provided an approach to discriminate the neighborhoods on the basis of changes in food availability.

## Methods

### Study area

We analyzed the Twin Cities Region (Minneapolis and St. Paul) of Minnesota (abbreviated as Twin Cities Region), an area of nearly three million people living in 186 communities across the seven counties of Anoka, Carver, Dakota, Hennepin, Ramsey, Scott, and Washington. The Twin Cities Region has developed several distinctive types of neighborhoods (e.g., active downtown, vibrant urban) [15]. In addition, from 1985 to 2010, the neighborhood environment in the Twin Cities Region became increasingly diverse in social composition and physical form [15]. Therefore, we expected that the Twin Cities Region would be an ideal case in which to observe temporal differences of, and changes in, the distribution of neighborhood food resources. Our study area included 2,083 census block groups defined in 2010 by the U.S. Census Bureau in the Twin Cities Region with diverse built environment and sociodemographic characteristics [17]. We used census block groups to operationalize neighborhoods. The census block group (approximate population of 1500) is the smallest unit for which data are available on built environment and sociodemographic measures. We excluded only two census block groups due to missing data.

### Relative availability of sit-down restaurants and supermarkets

We obtained food resource data from the D&B Duns Market Identifiers File (restaurant and food store Standard Industrial Classification categories; Dun & Bradstreet, Inc., Short Hills, NJ), a secondary commercial data source. We then classified the food resources according to primary eight-digit Standard Industrial Classification codes for data in years 1993, 2001, and 2011 (see Additional file 1: Table S1). We expected to compare business types from years 1990, 2000, and 2010; however, data for 1993, 2001 and 2011 were the only available Dun and Bradstreet business data.

Recent reports suggest that relative availability, i.e., particular proportions of various types of retail food outlets, may be more important to diet-related behaviors than the total number of outlets because relative availability offers residents competing options [18–20]. We chose to study the relative availability of sit-down restaurants and supermarkets. Sit-down restaurants such as ethnic food restaurants and seafood restaurants provide seating to eat instead of only food-to-go (either inside or drive-through). Although fast food restaurants have been linked with poor U.S. diet quality, evidence indicates that neither fast food nor sit-down restaurant were consistently more healthful [21–23]. Supermarkets were defined as large food stores that included chained or independent hypermarkets (greater than 100,000 square feet), supermarkets (66,000–99,000 square feet), and superstores (55,000–65,000 square feet) in the current study. In the U.S. context, supermarkets may have relatively more choices in and less expensive offerings of healthy food options compared with grocery stores and convenience stores, which are ubiquitous, smaller in size, and stocked with fewer or more expensive fresh and healthier food items compared with supermarkets [21–23]. We defined the relative availability of sit-down restaurants as the percent relative to total sit-down and fast food restaurants in a neighborhood (abbreviated below as percent of sit-down restaurants). We defined the relative availability of supermarkets as the percent relative to total supermarkets, grocery stores, and convenience stores in a neighborhood (abbreviated as percent of supermarkets below). We used a container-based approach to measure the relative availability of sit-down restaurants and supermarkets and defined the census block group as neighborhood. Therefore, our measure of the relative availability of sit-down restaurants and supermarkets was based on the evidence [24] that the types and distribution of food outlets in the neighborhood are associated with diet-related behavior. We used ArcGIS 10.3 to calculate the count of each type of food resource within each neighborhood in each observational year, and then we used the counts to calculate the percent of sit-down restaurants and supermarkets in STATA 14.0. When there was no sit-down or fast food restaurant, a constant of one was added to that case so that it remained in the analysis [13]. A previous study validated the D&B food resource data and showed that the matched rate of fast food restaurants may differ by various neighborhood characteristics such as income, race, and location (urbanized area, urban cluster and non-urban area as defined by the US Census Bureau) [25]. For example, if sit-down restaurants had a higher matched rate compared with fast food restaurants in low-income neighborhoods versus high-income neighborhoods in the D&B data, we risked exaggerating the gap in the numbers of sit-down restaurants relative to total sit-down restaurants and fast food restaurants between low-income and high-income neighborhoods. By using multiple dimensions to characterize neighborhood, we may partly address the varied matched rate issue because the lower matching rate raised by, for example, income is partly compensated by introducing mix use or population density factors to characterize neighborhoods jointly.

### Neighborhood type

To classify neighborhood type, we used a cluster analysis that included 13 built environment and sociodemographic characteristics in 1990. Because we did not have data for the same factors in 1993, we assumed that the 1990 built environment and sociodemographic data were a valid substitute for the 1993 data. In the following “Neighborhood built environment characteristics” and “Neighborhood sociodemographic characteristics” sections, we elaborated on the built environment and sociodemographic characteristics that we chose to generate the six types of neighborhoods. In “Cluster analyses” section we elaborated on the type of cluster analysis we employed to generate neighborhood type and techniques to examine the robustness of type classification. We did not generate the neighborhood type in 2001 and 2011 because our focus was to examine the change in neighborhood food availability over time based on the neighborhood type identified in the baseline year (1990).

### Neighborhood built environment characteristics

Neighborhood built environment characteristics included residential population density, employment population density, mix of land use, and percent of single-family housing in the neighborhood. These characteristics were used widely in the characterization of Western built environments [26–29]. We obtained the census population and land area size data in 1990, 2000, and 2006–2009 from the Census 1990, Census 2000, and the 2006–2009 American Community Survey. We used such data from the US Census Longitudinal Tract Database, which normalized the 1990, 2000, and 2006–2009 census data to the boundaries of census tracts in 2010. We interpolated the normalized census population density data for years from the census tract level to the census block-group level for years 1990, 2000, and 2010. We then measured residential population density as the total residential population divided by the total land area of the block group [30, 31], and we measured employment population density as the total employed civilian labor force aged 16 years and above divided by the total land area of the block group. These measures of total land area excluded large bodies of water and parks but included other land uses such as commercial lands and roadways. We obtained data on categories and areas of different types of land uses for the creation of land use mix and percent of single-family housing from the GIS-based current land-use map in 1990, 2000, and 2010 from the Minneapolis Metropolitan Council. We measured the mix of land use using the 3-tier land use entropy equation, with three land use categories (residential, employment and retail) to calculate mix of land use in the block group [32]. Land use entropy ranges in value from zero (total homogeneity, with a single land use present) to 1 (maximum heterogeneity, with an even distribution across the three land uses). We defined the percent of single-family housing as the number of single-family housing units divided by the total number of single-family and multi-family housing units.

### Neighborhood sociodemographic characteristics

Neighborhood sociodemographic characteristics included percent of population aged under 14, aged 15–29, 30–44, 45–64, and aged 65 or above according to working age, percent of education of college or above, percent of white race, percent of black race, and median household income. We retrieved all the census sociodemographic characteristics in 1990, 2000, and the 2006–2009 American Community Survey of the U.S. Census Bureau from the US Census Longitudinal Tract Database. We then interpolated the normalized census sociodemographic characteristics data from the census tract level to the census block-group level.

### Cluster analyses

Others have used data reduction techniques such as Principal Component Analysis and factor analysis [10, 33] to group variables and generate a composite index or quantiles to classify neighborhoods into different types. Conversely, instead of variables, we used K-means cluster analysis, a partitioning approach, to group observations (i.e., neighborhoods) by data-mining techniques. Using these methods, we measured the intrinsic relationship between neighborhood characteristics based on a Euclidean K-means clustering algorithm. We first transformed each 1990 built environment and sociodemographic variable into a z-score to achieve more comparable scales and ranges; otherwise, variables with large ranges might have weighed heavier in the analysis than variables with small ranges [34]. We then used the transformed data to perform partition cluster analyses within the 13 built environment and sociodemographic characteristics, using K-means in Stata 14.0. To assess goodness of fit and select a final number of clusters we used three statistical approaches, Gap Statistic Method, Average Silhouette Method and Elbow Method [34]. These three methods recommended six, seven, and six or seven clusters, respectively (Additional file 2: Figures S1–S3). We compared the associated cluster statistics between six-cluster and seven-cluster solutions. Compared with the six-cluster solution, the seven-cluster solution split the six-cluster suburban edge neighborhoods (n = 672) into two subclusters, one (n = 416) included 414 neighborhoods from the six-cluster suburban edge cluster, and the other (n = 397) included 253 neighborhoods from the six-cluster suburban edge cluster plus 104 neighborhoods from the six-cluster aging suburb cluster. As the two subclusters did not differ significantly in neighborhood characteristics such as residential population density, employment population density, and percent of single-family housing, we chose the six-cluster solution.

### Covariates

Sit-down restaurants tend to be located in high density neighborhoods because of walkability and the cozy atmosphere offered by urban environments [35, 36]. Sit-down restaurants and supermarkets are less likely to be located in Black or poor neighborhoods [36–40]. In addition, highly restrictive ordinances may limit the presence of sit-down restaurants near particular land uses, such as single-family housing, partly because of perceptions that sit-down restaurants may have local deleterious impacts such as traffic, noise, and possible promotion of unlawful behaviors [41, 42]. On the basis of such reports, we incorporated the four variables, residential/employment population density, median household income, percent of white race, and percent of single-family housing as covariates into the models. For those four covariates, to represent the changes in neighborhood characteristics during that period, we added to our models four time-varying variables, which were the changes in residential/employment population density, median household income, percent of white race, and percent of single-family housing from 1990. We calculated changes in residential population density, median household income, percent of white race, and percent of single-family housing by the same method that we used to calculate changes in employment population density. We used the changes in employment population density and residential population density in the sit-down restaurant and supermarket models, respectively. Adding such change variables was necessary because we measured neighborhood type only for 1993, which could not be used to explain the change in percent of sit-down restaurants and supermarkets between 1993 and 2011.

Sit-down restaurant purveyors may prefer to locate their restaurants in neighborhoods that already have a large number of restaurants to draw customers who may seek variety [43, 44]. Therefore, we added the total number of sit-down restaurants and fast food restaurants as one of covariates in the sit-down restaurant model. However, supermarket purveyors may not prefer to locate in neighborhoods that already have a large number of different types of food stores because competition may reduce the likelihood of customers who tend to prefer to shop at a specific outlet [45]. Therefore, we added the total number of supermarkets, grocery stores and convenience stores as one of the covariates in the supermarket model.

### Statistical analyses

All descriptive analyses and multivariable models were performed using Stata 14.0 (StataCorp, College Station, TX).

#### Descriptive statistics

We calculated means and standard deviations (for continuous variables) of neighborhood built environment characteristics, neighborhood sociodemographic characteristics, and the relative availability of sit-down restaurants and supermarkets in the neighborhood in 1990/1993, 2001 and 2011. We used one-tailed Student’s t-test and Kruskal–Wallis H test to test for statistically significant differences in means and medians for continuous variables.

#### Relationship between neighborhood type and relative availability of sit-down restaurants and supermarkets

We used multivariable linear mixed effects regression models to estimate the associations between neighborhood type in 1993 and the percent of sit-down restaurants and percent of supermarkets in 1993, 2001, and 2011 (n = 2083). These models appropriately accounted for repeated measurements over time within each neighborhood. Specifically, one neighborhood in 1993 had many similarities compared to the same neighborhood in 2001 and 2011, which may have violated the principal of independently and identically distributed observations. To address the “repeated-measurement” feature of the data, we implemented mixed effects regression models for the percent of sit-down restaurants and percent of supermarkets. We modeled the percent of sit-down restaurants/supermarkets in each neighborhood as a function of neighborhood type in 1993, the time elapsed in years from 1993, the term for the interaction of neighborhood type in 1993 with elapsed time, and the time-varying covariates, which we denoted as baseline-change models [46]. We performed baseline-change analysis to assess how neighborhood characteristics (as measured by neighborhood type) at the baseline year modified the effect of time on the relative availability of sit-down restaurants and supermarket. If “neighborhood type at the baseline year” failed to modify the effect of time on the relative availability of sit-down restaurants and supermarkets, then the increase rates in the relative availability of sit-down restaurants and supermarkets should be the same across the baseline-year neighborhood type. Further, we employed post-estimated linear contrasts based on the results of same models, which enabled us to compare the relative availability of sit-down restaurants and supermarkets across neighborhood type in each observational year. We included random intercepts for each neighborhood in the sit-down restaurant and supermarket models to enable responses to vary within neighborhoods. Because census block groups were is a small area in dense areas, we tested whether our results were sensitive with respect to different measures of relative availability of sit-down restaurants and supermarkets based on census tract as well as census place (i.e., city or town). We incorporated a ‘change in neighborhood type over time’, but models with this change variable failed to converge, partly because approximately 68% of neighborhoods did not change type over time. Thus, to capture neighborhood changes we added change variables for residential and/or employment population density and neighborhood sociodemographic variables specific to each outcome.

## Results

### Descriptive statistics

Compared with 1993, in 2011, the percent of sit-down restaurants and supermarkets in the study area increased 10.1 and 3.3 percentage points, respectively (Table 1). Our study area’s population in 2011 (compared with 1993) tended to be older (45–64 or 65 or above), more non-white, more college educated or higher, and having higher household incomes. The study area had a greater population density, greater mix of land use, and greater percent of single-family housing in 2011 compared with 1993.

**Table 1:** Selected characteristics of neighborhoods in years 1993, 2001 and 2011, Twin Cities Region

Neighborhood characteristic	1993 ^a^	2001	2011	Change ^b^	P value ^c^
Number of observations (neighborhoods)	2083	2083	2083	–	–
Relative availability of sit-down restaurants and supermarkets					
Percent of sit-down restaurants^d^, mean (SD)	16.1 ± 33.1	22.7 ± 36.4	26.2 ± 36.8	10.1 ± 41.4	< 0.05
Percent of supermarkets^e^, mean (SD)	2.0 ± 11.9	2.4 ± 12.9	5.3 ± 19.6	3.3 ± 20.1	< 0.05
Built environment characteristics					
Residential population density, 1000 person/km^2^, median (IQR)	1.2 (1.6)	1.2 (1.3)	1.3 (1.3)	0.0 (0.5)	< 0.05
Employment population density, 1000 person/km^2^, median (IQR)	0.6 (0.8)	0.7 (0.7)	0.7 (0.7)	0.0 (0.1)	< 0.05
Mix of land use ^f^, median (IQR)	45.0 (48.0)	51.3 (49.7)	57.0 (46.0)	8.7 (20.4)	< 0.05
Percent of single-family housing^g^, median (IQR)	68.5 (65.4)	73.2 (64,9)	94.9 (36.8)	16.3 (27.0)	< 0.05
Total sit-down restaurants and fast food restaurants, median (IQR)	0.0 (1.0)	0.0 (2.0)	1.0 (2.0)	1.3 (3.2)	< 0.05
Total supermarkets, grocery stores and convenience stores, median (IQR)	0.0 (1.0)	1.0 (1.0)	1.0 (2.0)	0.2 (1.2)	< 0.05
Sociodemographic characteristics					
Age, mean (SD)					
Percent of population under 14	22.2 ± 6.4	21.3 ± 6.8	19.9 ± 6.0	− 2.3 ± 4.8	< 0.05
Percent of population 15–29	23.7 ± 7.3	20.9 ± 7.8	21.6 ± 8.4	− 2.1 ± 4.6	< 0.05
Percent of population 30–44	27.2 ± 4.7	26.0 ± 4.8	21.9 ± 4.5	− 5.3 ± 5.0	< 0.05
Percent of population 45–64	16.9 ± 5.0	21.2 ± 5.4	25.8 ± 5.7	8.9 ± 5.8	< 0.05
Percent of population 65 or above	9.9 ± 6.6	10.5 ± 7.3	10.8 ± 6.0	0.9 ± 5.7	< 0.05
Percent of population with education level of college or above, mean (SD)	57.6 ± 15.2	66.1 ± 14.9	68.1 ± 14.3	10.5 ± 8.6	< 0.05
Race, median (IQR)					
Percent of white race	96.0 (5.0)	90.6 (13.0)	87.0 (16.0)	− 10.8 (11.6)	< 0.05
Percent of black race	1.0 (3.0)	2.9 (5.1)	4.0 (9.0)	4.1 (7.2)	< 0.05
Median household income^h^, $1000, mean (SD)	38.2 ± 12.5	40.5 ± 15.1	37.1 ± 14.5	0.4 ± 7.5	< 0.05
Time elapsed from 1993, year, mean (SD)	0 ± 0	8 ± 0	18 ± 0	18 ± 0	–

IQR, interquartile range; SD, standard deviation

^a^Because we did not have neighborhood built environment and sociodemographic data for 1993, we assumed that data for 1990 would be valid substitutes for the missing 1993 data

^b^Change in neighborhood characteristics from year 1993 to 2011

^c^P value for one-tailed Student’s t-test of difference in means and Kruskal–Wallis H test of difference in medians from years 1993 and 2011

^d^Percent of sit-down restaurants relative to total sit-down restaurants and fast food restaurants

^e^Percent of supermarkets relative to total supermarkets, grocery stores and convenience stores

^f^The mix of land use was measured by examining three land use categories at the census block group (residential, employment and retail)

^g^Percent of single-family housing relative to total single-family and multi-family housings

^h^The median household incomes in 1993 and 2001 were adjusted for inflation to compare with that in 2011

### Results from cluster analyses: neighborhood type (Year 1993)

The six robust neighborhood types that we defined by the final cluster solution represented non-overlapping groupings of Twin Cities Region neighborhoods based on built environment and sociodemographic attributes in 1993 (the first observational year). These clusters included: cluster 1—high-density urban core; cluster 2—low-income, non-white inner city; cluster 3—urban; cluster 4—aging suburb; cluster 5—high-income suburb; and cluster 6—suburban edge.

These clusters were labeled based on their most prominent built environment and sociodemographic characteristics in 1993 (see Additional file 3: Table S1). Compared with most of the other clusters, cluster 1, “high-density urban core”, had relatively greater levels of residential and employment population densities, a greater mix of land use, comparatively lower percent single-family housing, comparatively higher percent population aged 15–29, and comparatively lower percent population aged under 14. Cluster 2, “low-income, non-white inner city”, had moderate-to-high residential and employment population densities and comparatively higher percent non-white race population, relatively lower level of median household income and comparatively lower percent population with a college education or above. Cluster 5 and Cluster 6, “high-income suburb” and “suburban edge”, had relatively lower levels of residential and employment population densities, lower degrees of mix of land use, and relatively greater levels of median household income. Cluster 3 (“urban”) and Cluster 4 (“aging suburb”) had moderate levels of almost all neighborhood features, except for a greater degree of mix of land use and comparatively higher percent population aged 65 or above.

**Fig. 1:**
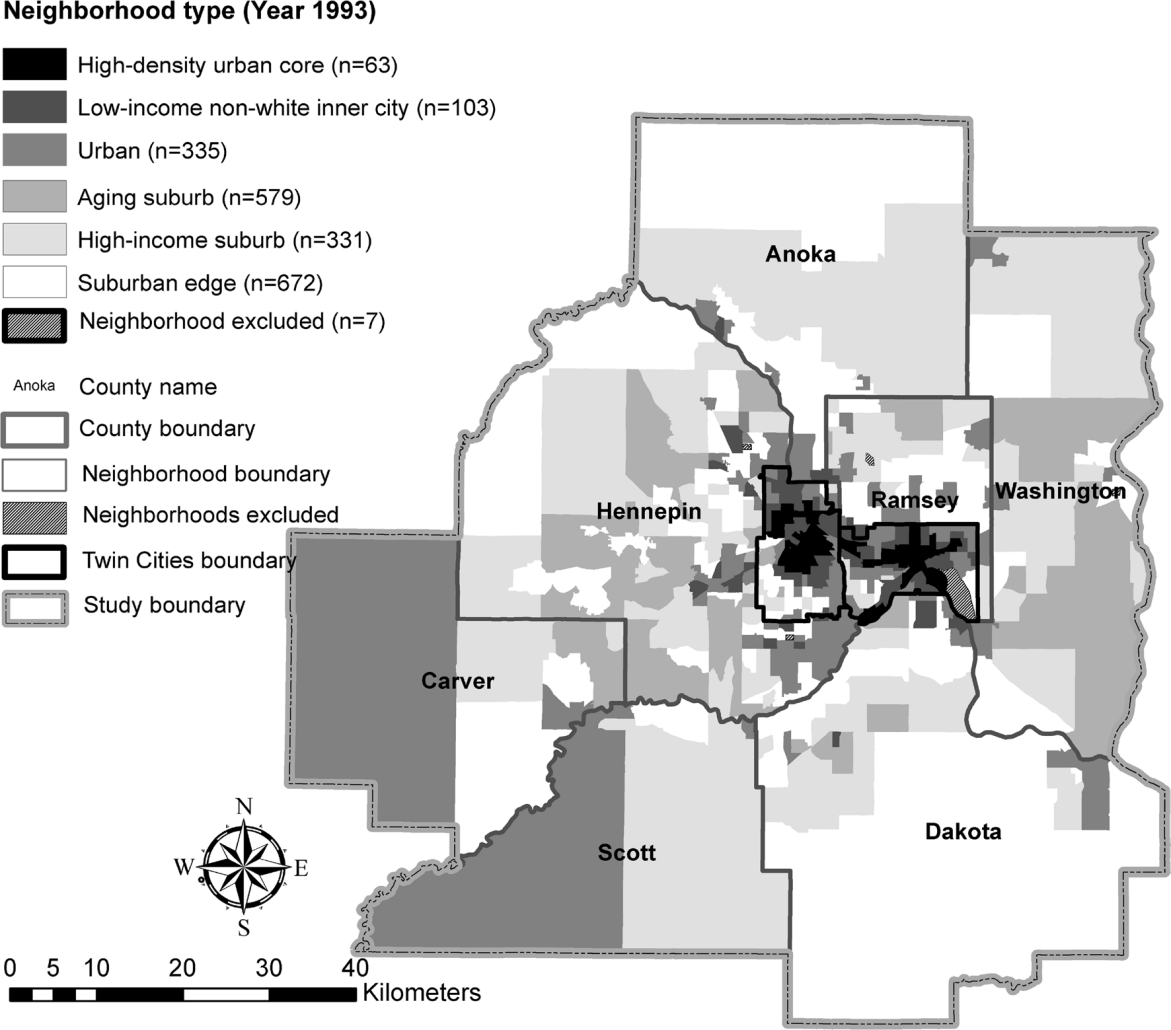
Neighborhood types in 1993 in the Twin Cities Region of Minnesota

Figure 1 shows that the high-density urban core (abbreviated as urban core) and low-income, non-white inner city (abbreviated as inner city) neighborhoods were tightly clustered in a small segment within the municipal boundaries of the Twin Cities. Urban and aging suburb neighborhoods comprised those transitional areas located between the urban core or inner city neighborhoods and the suburban areas. Another small grouping of aging suburb and high-income extended into the counties of Carver and Scott and the county of Washington, respectively. The generated clusters reflected comprehensive but distinguishable physical and sociodemographic environments.

### Relationship between neighborhood type and relative availability of sit-down restaurants and supermarkets

**Fig. 2:**
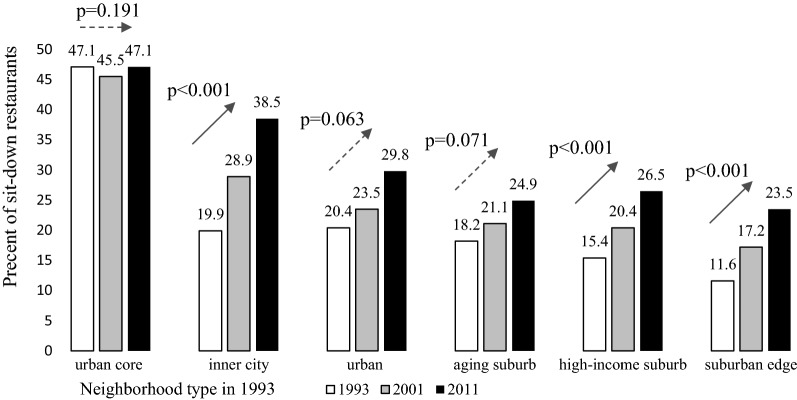
Estimated mean** a** of percent of sit-down restaurants by six types of neighborhoods** b**.** a** Multivariable mixed effects regression modeling percent of sit-down restaurants relative to total sit-down restaurants and fast food restaurants in each neighborhood as a function of neighborhood type in 1993, time elapsed since 1993, interaction between neighborhood type in 1993 and time elapsed, changes in employment population density, median household income, percent of white and percent of single-family housing since 1993, total sit-down restaurants and fast food restaurants and a random intercept for each neighborhood.** b** Derived from cluster analysis of block-group level data in 1993: percent of age under 14, age aged 15–29, 30–44, 45–64, and aged above 65, percent of education of college and above, percent of white, percent of black, median household income, residential population density, employment population density, mix of land use and percent of single-family housing

**Fig. 3:**
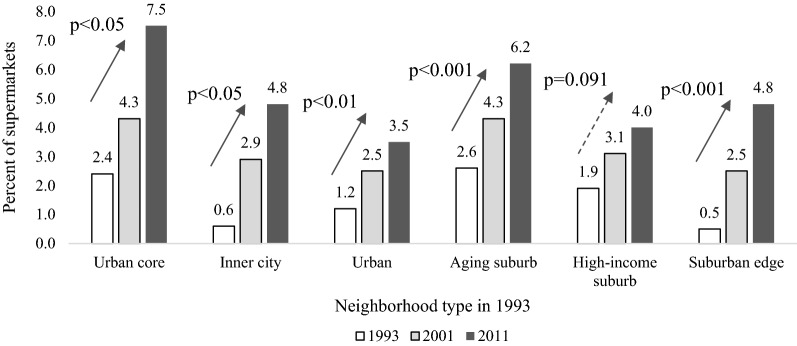
Estimated mean** a** of percent of supermarkets relative to total supermarkets, grocery stores and convenience stores by six types of neighborhoods** b**: Twin Cities Region, 1993–2011.** a** Multivariable mixed effects regression modeling percent of supermarkets relative to total supermarkets, grocery stores and convenience stores in each neighborhood as a function of neighborhood type in 1993, time elapsed since 1993, interaction between neighborhood type in 1993 and time elapsed, changes in residential population density, median household income, percent of white and percent of single-family housing since 1993, total supermarkets, grocery stores and convenience stores and a random intercept for each neighborhood.** b** Derived from cluster analysis of block-group level data in 1993: percent of age under 14, aged 15–29, 30–44, 45–64, and aged above 65, percent of education of college or above, percent of white, percent of black, median household income, residential population density, employment population density, mix of land use and percent of single-family housing

The results of multivariable linear mixed effects regression models suggest that urban core (p = 0.191), urban (p = 0.063), and aging suburb (p = 0.071) did not experience a significant increase (at a 0.05 statistical level) in the percent of sit-down restaurants (Fig. 2). High-income suburb (p = 0.091) did not experience a significant increase in the percent of supermarkets (Fig. 3) between 1993 and 2011. The coefficients of − 1.15, − 0.53, and − 0.58 in the same sit-down restaurant model suggest that inner city neighborhoods experienced a greater increase in the percent of sit-down restaurants compared with urban core, urban, and aging suburb between 1993 and 2011 (Additional file 4: Table S1).

The results in Tables 2, 3, and 4 were derived from the same models as Figs. 2, 3 and Additional file 4: Tables S1, S2. Tables 2, 3, and 4 shows the post-estimated linear contrasts of percent of sit-down restaurants and percent of supermarkets in the neighborhood by year and for each neighborhood type pair from the multivariable linear mixed effects regression models. Urban core neighborhoods had a higher percent of sit-down restaurants (by 26.61–35.48 percentage points) compared with the other five types of neighborhoods in 1993 (Table 2). The coefficient of − 27.13 in 1993 in the sit-down restaurant model (Table 2) suggested that the percent of sit-down restaurants in inner city neighborhoods was 27.13% lower than that of urban core neighborhoods in 1993. And the confidence interval of − 28.62 and − 25.63 indicated that we had 95% confidence that the actual difference in the percent of sit-down restaurants between inner city and urban core neighborhoods fell between 25.63 and 28.62 in 1993. We did not observe any detectible differences in the percent of supermarkets between urban core and other types of neighborhoods for 1993 (Table 2). For 2001, we observed more differences in percent of sit-down restaurants and supermarkets by neighborhood type. Inner city neighborhoods had a higher percent of sit-down restaurants (by 5.38 percentage points) than did urban neighborhoods; aging suburb neighborhoods had slightly more supermarkets (1.59–1.78 percentage points) compared with the urban and suburban edge neighborhoods (Table 3). In 2011, the differences in the percent of sit-down restaurants between urban core and other neighborhoods decreased to between 8.52 and 23.57, whereas the differences in the percent of sit-down restaurants between inner city and other neighborhoods (aging suburb, high-income suburb and suburban edge neighborhoods) increased to between 8.7 and 15.05 (Table 4), compared to the difference in the percent of sit-down restaurants between 1.69 and 8.36 in 1993 and between 5.38 and 11.72 in 2001. We performed a multicollinearity test and generated values for a variation inflation factor greater than 10 for two variables (baseline neighborhood type and interaction term for baseline neighborhood type and year). Because baseline neighborhood type was statistically significant in the sit-down model but not the supermarket model even with exclusion of the interaction term, we concluded that the high correlation between baseline neighborhood type and the interaction term did not obscure interpretation of the parameter estimate of baseline neighborhood type. The values of the variation inflation factor were less than five for covariates other than baseline neighborhood type and the interaction term. We used the performance package in R i386 3.5.3 to test the magnitude of multicollinearity in the mixed effects models.

**Table 2:** Contrast^a^ of percent of sit-down restaurants^b^ and supermarkets^c^ for each neighborhood type^d^ pair in 1993

	Urban core	Inner city	Urban	Aging suburb	High-income suburb	Suburban edge
Sit-down restaurant model: estimated beta (95% confidence interval)						
Urban core	–	–	–	–	–	–
Inner city	*− 27.13* *(− 28.62, − 25.63)*	–	–	–	–	–
Urban	*− 26.61* *(− 29.45, − 23.77)*	0.51 (− 1.71, 2.74)	–	–	–	–
Aging suburb	*− 28.82* *(− 30.25, − 27.39)*	*− 1.69* *(− 2.82, − 0.57)*	*− 2.21* *(− 3.29, − 1.11)*	–	–	–
High-income suburb	*− 31.69* *(− 32.90, − 30.48)*	*− 4.57* *(− 5.52, − 3.61)*	*− 5.08* *(− 6.37, − 3.78)*	*− 2.87* *(− 3.56, − 2.18)*	–	–
Suburban edge	*− 35.48* *(− 36.50, − 34.47)*	*− 8.36* *(− 9.15, − 7.56)*	*− 8.87* *(− 9.81, − 7.93)*	*− 6.67* *(− 7.18, − 6.14)*	*− 3.79* *(− 4.31, − 3.27)*	–
Supermarket model: estimated beta (95% confidence interval)						
Urban core	–	–	–	–	–	–
Inner city	− 1.87 (− 6.24, 2.50)	–	–	–	–	–
Urban	− 1.41 (− 5.33, 2.51)	0.46 (− 2.67, 3.59)	–	–	–	–
Aging suburb	− 0.18 (− 4.11, 3.75)	1.69 (− 1.39, 4.77)	1.23 (− 0.66, 3.12)	–	–	–
High-income suburb	− 0.58 (− 4.78, 3.63)	1.30 (− 2.07, 4.66)	0.84 (− 1.38, 3.06)	− 0.39 (− 2.30, 1.51)	–	–
Suburban edge	− 1.68 (− 5.83, 2.46)	0.19 (− 3.08, 3.46)	− 0.27 (− 2.29, 1.75)	− 1.50 (− 3.13, 0.13)	− 1.11 (− 2.94, 0.73)	–

Italics indicates significant difference in percent of sit-down restaurants or percent of supermarkets across neighborhood type at the 0.05 significance level

^a^Multivariable linear mixed effects regressions modeling the percent of sit-down restaurants relative to total sit-down restaurants and fast food restaurants and percent of supermarkets relative to total supermarkets, grocery stores and convenience stores as functions of neighborhood type in 1993, time elapsed since 1993, interaction between neighborhood type in 1993 and time elapsed, change in employment population density (sit-down restaurant model only), change in residential population density (supermarket model only), median household income, percent of white race and percent of single-family housing since 1993, total sit-down restaurants and fast food restaurants (sit-down restaurant model only), and total supermarkets, grocery stores and convenience stores (supermarket model only) and a random intercept for each neighborhood

^b^Percent of sit-down restaurants relative to total sit-down restaurants and fast food restaurants in the neighborhood

^c^Percent of supermarkets relative to total supermarkets, grocery stores and convenience stores in the neighborhood

^d^Derived from cluster analysis of block-group level data from 1993: percent of population aged under 14, aged 15–29, 30–44, 45–64, and aged above 65, percent of education of college or above, percent of white race, percent of black race, median household income, residential population density, employment population density, mix of land use and percent of single-family housing

**Table 3:** Contrast^a^ of percent of sit-down restaurants^b^ and supermarkets^c^ for each neighborhood type^d^ pair in 2001

	Urban core	Inner city	Urban	Aging suburb	High-income suburb	Suburban edge
Sit-down restaurant model: estimated beta (95% confidence interval)						
Urban core	–	–	–	–	–	–
Inner city	*− 16.66* *(− 18.97, − 14.35)*	–	–	–	–	–
Urban	*− 22.04* *(− 25.49, − 18.60)*	*− 5.38* *(− 8.05, − 2.71)*	–	–	–	–
Aging suburb	*− 24.45* *(− 26.35, − 22.55)*	*− 7.79* *(− 9.25, − 6.33)*	*− 2.41* *(− 3.74, − 1.08)*	–	–	–
High-income suburb	*− 25.10* *(− 26.96, − 23.25)*	*− 8.44* *(− 9.85, − 7.03)*	*− 3.06* *(− 4.66, − 1.46)*	− 0.65 (− 1.58, 0.28)	–	–
Suburban edge	*− 28.38* *(− 30.00, − 26.75)*	*− 11.72* *(− 12.97, − 10.46)*	*− 6.33* *(− 7.53, − 5.14)*	*− 3.92* *(− 4.65, − 3.20)*	*− 3.27* *(− 4.08, − 2.46)*	–
Supermarket model: estimated beta (95% confidence interval)						
Urban core	–	–	–	–	–	–
Inner city	− 2.02 (− 5.51, 1.48)	–	–	–	–	–
Urban	− 2.09 (− 5.34, 1.15)	− 0.78 (− 2.58, 2.42)	–	–	–	–
Aging suburb	− 0.32 (− 3.66, 3.03)	1.70 (− 0.82, 4.22)	*1.78* *(0.26, 3.29)*	–	–	–
High-income suburb	− 1.91 (− 5.52, 1.70)	0.11 (− 2.66, 2.88)	0.19 (− 1.63, 2.00)	0.25 (− 1.40, 1.91)	–	–
Suburban edge	− 1.84 (− 5.41, 1.74)	0.18 (− 2.52, 2.88)	0.19 (− 1.63, 2.00)	*− 1.59* *(− 3.13, − 0.05)*	*− 1.52* *(− 2.84, − 0.21)*	–

Refer to the legends in Table 2

**Table 4:** Contrast^a^ of percent of sit-down restaurants^b^ and supermarkets^c^ for each neighborhood type^d^ pair in 2011

	Urban core	Inner city	Urban	Aging suburb	High-income suburb	Suburban edge
Sit-down restaurant model: estimated beta (95% confidence interval)						
Urban core	–	–	–	–	–	–
Inner city	*− 8.52* *(− 11.99, − 5.05)*	–	–	–	–	–
Urban	*− 17.22* *(− 23.24, − 11.21)*	*− 8.70* *(− 13.35, − 4.06)*	–	–	–	–
Aging suburb	*− 22.16* *(− 24.92, − 19.40)*	*− 13.64* *(− 15.73, − 11.55)*	*− 4.94* *(− 7.14, − 2.73)*	–	–	–
High-income suburb	*− 20.52* *(− 23.28, − 17.75)*	*− 12.00* *(− 14.03,− 9.97)*	− 3.29 (− 6.02, 0.57)	*1.64* *(0.31, 2.98)*	–	–
Suburban edge	*− 23.57* *(− 26.56, − 20.58)*	*− 15.05* *(− 17.33, − 12.77)*	*− 6.34* *(− 8.50, − 4.19)*	*− 1.41* *(− 2.61, − 0.21)*	*− 3.05* *(− 4.46, − 1.64)*	–
Supermarket model: estimated beta (95% confidence interval)						
Urban core	–	–	–	–	–	–
Inner city	− 2.19 (− 6.76, 2.37)	–	–	–	–	–
Urban	− 2.95 (− 7.13, 1.24)	− 0.75 (− 3.98, 2.48)	–	–	–	–
Aging suburb	− 0.48 (− 4.72, 3.76)	1.71 (− 1.47, 4.90)	*2.46* *(0.50, 4.43)*	–	–	–
High-income suburb	− 3.57 (− 8.04, 0.89)	− 1.38 (− 4.80, 2.04)	− 0.63 (− 2.98, 1.72)	*− 3.09* *(− 5.14, − 1.05)*	-	–
Suburban edge	− 2.03 (− 6.39, 2.32)	0.16 (− 3.10, 3.42)	0.91 (− 1.16, 2.98)	− 1.55 (− 3.26, 0.15)	1.54 (− 0.38, 3.46)	–

Refer to the legends in Table 2

### Sensitivity testing

Additional file 4: Tables S1, S2 contain regression results using the census tract and place to measure food availability. Tract models generated similar results to the main results based on block group. But place models showed inconsistencies, particularly for the sit-down restaurant model. Urban and aging suburb neighborhoods experienced lower increases in the percent of sit-down restaurants than inner city in the block group and tract models, whereas we failed to observe such a difference in the place model. Similarly, urban core had higher percent of sit-down restaurants than inner city in 1993 in the block group and tract models, but the place model did not show such a difference. The differences between the block group, tract and place models suggested that the measure of relative availability was sensitive to spatial unit. Because the size of a census block group was not always small (varying from 0.04 in the urban core to 154.19 km^2^ in the suburban edge with median and interquartile range values of 0.88 and 1.63) and increased with the distance to urban core increases, our measure of relative food availability was a reasonable small-area measure.

## Discussion

The objective of this study was to assess the relationship between neighborhood characteristics and neighborhood food availability. We proposed that neighborhoods are composed of interrelated sociodemographic and built environment factors, and such factors jointly affect the distribution and type of food outlets. We recognized that analyses may be confounded by correlations among neighborhood features; thus, we used cluster analysis to identify six types of neighborhoods in the Twin Cities Region of Minnesota that reflected distinct combinations of built environment with sociodemographic features. Then, we examined the association between neighborhood type in the baseline year and neighborhood food availability as measured by the relative availability of types of food outlets relative to other types of food outlets. Our results indicated an increasingly varied distribution of restaurants and food stores by neighborhood type over time. Our findings contribute to a growing literature on the associations between the multifaceted composition of the built environment, sociodemographic features, and the distribution of food resources. Our study demonstrates the need to use methods such as cluster analysis to characterize neighborhoods on the basis of diverse sets of characteristics. Using this approach, we identified neighborhoods that experienced different changes in food availability over time. Simple neighborhood characterization by sociodemographic factors alone may mask these important complexities.

Our neighborhood types were not spatially clustered into homogeneous regions but, instead, were distributed across the Twin Cities Region. For example, the municipal boundaries of the Twin Cities did not contain only urban core and inner city neighborhoods but also included urban and aging suburbs. Similarly, aging suburbs and high-income neighborhoods extended to the boundaries of the region; thus, they were typically farther from the city center. Therefore, our results support the work of others who noted a recent blending of built environment and sociodemographic characteristics, resulting in reduced demarcation between the central city and its outlying suburban areas [47, 48]. Because both the central cities and the outlying areas in metropolitan U.S. are becoming more diverse in both urban form and social composition [48, 49], reliance on single constructs of neighborhoods, such as population density or distance to central business district, may not adequately capture the complexity of neighborhood types.

The inner city neighborhoods had a greater relative availability of sit-down restaurants compared with other neighborhoods in 2001 and 2011; we failed to observe such differences in 1993. This suggested that inner city neighborhoods became relatively more appealing to proprietors of sit-down restaurants and, perhaps, less appealing to owners of fast food restaurants. Although inner city neighborhoods consistently had the lowest household income during the observational period (Additional file 3: Tables S1–S3), inner city neighborhoods had greater spatial access to sit-down restaurants than other neighborhoods in 2011. In another of our studies [24], we observed a positive association between the GIS-measured count of neighborhood sit-down restaurants and the frequency of using neighborhood sit-down restaurants in non-rural areas in four metropolitan areas including the Twin Cities Region. Thus, perhaps indicating that greater availability of sit down restaurants translates to their greater use. Another possibility is that more sit-down restaurants in the inner city did not necessarily mean that inner city people ate at those venues. Inner cities increasingly serve as one part of a social hub in a region, and many people who visit inner cities and use sit-down restaurants may be from the surrounding suburban or rural area [50]. That is, it is possible that the draw of big cities, which include cultural amenities, entertainment and other facilities leads to an increase in sit-down restaurants to serve employees and tourists, as seen in New York, NY [51], Houston, TX [52], Washington, DC [53], and metropolitan areas [54]. Employment population density is the number of employed persons (divided by the area size of the neighborhood) who work but who do not necessarily reside in the neighborhood. With increases in employment population density, we observed an increase in relative availability of sit-down restaurants (Additional file 4: Table S1). This positive association between the change in employment population density and relative availability of sit-down restaurants suggests the possibility that availability of neighborhood sit-down restaurants may relate more to the employment sector than neighborhood residents. During our study period, U.S. inner cities transitioned from goods production sectors toward relatively place-bound service sector industries [48, 55], which includes restaurants [56, 57]. Lester et al. [57] observed that, in twenty U.S. inner cities between 1990 and 2000, jobs in retail services replaced jobs lost in goods-producing industries. Retail- and service-dominated neighborhoods may have provided a complementary environment for clustering of restaurants, food stores and other retail options [55]. Improvements in transportation and landscaping may have created a more spatially accessible and/or walkable features that attract service and retail options [58, 59]. During the study period, the Twin Cities experienced improvements in light rail, the park system, and new sports stadiums [60]. Future research should evaluate the process by which consumer visits to sit-down restaurants in cities and how consumer demand related to growth in restaurants. Identifying the nature and types of food stores in neighborhoods has potential implications on the health of residents. Mezuk et al. [61] found that neighborhoods with high proportions of “health-harming” stores (for example, fast-food outlets, bars, or pubs as listed by the authors) had higher community-level prevalence of Type II diabetes. Although sit-down restaurants are not necessarily “health-harming” outlets, many investigators [21–23] have found that sit-down restaurants are no more likely to sell healthy foods than fast food restaurants.

The percent of sit-down restaurants in urban core neighborhoods was stable during the observational period [50, 62]. We found no increase in the percent of sit-down restaurants in the urban core. Urban cores already had a high percent of sit-down restaurants in 1993 (46.9% in Fig. 2). This constant percent implies a “saturated” urban core with respect to the relative availability of sit-down restaurants. The unchanged relative availability of sit-down restaurants paralleled an increase in the well-educated population who predominately lived in urban core neighborhoods over the follow up period. This finding was similar to observations for Houston, TX [52], which also showed an increase in the well-educated population in urban core neighborhoods.

The Twin Cities Region experienced multiple different economic conditions during the period of our study: economic expansion (1993–2007), economic recession (2007–2009), and economic recovery (2009–2011) [63]. Nevertheless, the average percent of sit-down restaurants increased in the Region from 16% in 1993 to 23% in 2001 to 26% in 2011. We assumed that the percent of sit-down restaurants would change little or even decrease during economic recession (2007–2009) because demand for meals from sit-down restaurants is price-elastic [64]. Our results seem to contradict that assumption. The increase in the percent of sit-down restaurants in the Twin Cities Region, particularly from 2006 to 2011, was perhaps greater than that of the nation at large. Similarly, Richardson’s study [65] implied a relatively stable percent of sit-down restaurants between 1985 and 2006 for four regions in the U. S. (see Table 4 in her work), Birmingham, AL; Chicago, IL; Minneapolis, MN; and Oakland, CA. Our results suggest that convenience, sociodemographic characteristics, and macroeconomic forces such as the business cycle, instead of only relative prices and income [66, 67], may be why we still saw a significant increase in the percent of sit-down restaurants instead of fast food restaurants in most Twin Cities neighborhoods.

We also found more varied distribution of food stores across neighborhoods in 2001 and 2011 that we did not see for 1993. Specifically, aging suburb neighborhoods had a greater percent of supermarkets (i.e., fewer percent of grocery stores and convenience stores) than did the urban and high-income suburb neighborhoods in 2001 and 2011, but not in 1993. Such differences were driven largely by the great increase in the number of grocery stores and convenience stores in the high-income and suburban edge neighborhoods in comparison with increases in numbers of aging suburb supermarkets. The higher percent of grocery and convenience stores in urban and high-income neighborhoods may compound barriers to accessing healthful foods, if such foods are less available in grocery and convenience stores [68]. Small food stores may offer an abundance of less nutritious foods such as sugar-sweetened beverages, salty snacks, and candy, and the prices of fresh foods are likely to be more expensive than in US supermarkets [3–5]. Thus, it is possible that the higher percent of grocery and convenience stores in urban and high-income neighborhoods may mean that less nutritious foods are abundantly available in such neighborhoods [33]. However, we found that an increased percent of supermarkets was associated with a smaller increase (or more rarely a decrease) in the percent of single-family housing units (see Additional file 4: Table S2). These largely incompatible land uses—single-family housing and supermarkets—may have opened opportunities for urban planners to use regulatory tools (e.g., zoning) to introduce targeted food stores into the neighborhoods. These regulatory tools could side-step concerns/requirements such as intrusive light [69], sufficient parking [41], or increased traffic, thereby avoiding resistance to introducing a supermarket into neighborhoods with large increase of single-family housing.

Researchers are increasingly using complex data-rich methods to define and distinguish neighborhoods. In this vein, we chose sociodemographic and built environment variables that potentially related to the distribution of food outlets to define neighborhood types. Because of the wide application of Geographic Information System techniques, it is feasible for researchers to generate spatial data and combine abundant location data for use in clustering analyses. Therefore, although we examined only one large metropolitan (geographical) region, our method to assess associations between this complex group of neighborhood characteristics and food availability is generalizable. We suggest cluster analysis to characterize neighborhoods, given their complexity with greater urbanicity [13]. For example, our findings pointed to nine subclusters of low-income non-white neighborhoods and three to nine low-income non-white block groups within each subcluster. These distributions suggest that low-income non-white neighborhoods were not necessarily located within the central city of the region. If we used simple approaches to classify areas, such as distance to the census tract that contains the city hall, we may have failed to notice that some low-income non-white neighborhoods were surrounded by aging suburb and high-income suburb neighborhoods in the Hennepin County, which could have implications for food availability. Thus, patterning techniques such as the cluster analysis technique we used in this study adds a meaningful approach to characterizing neighborhoods.

Our study has several caveats. First, the Twin Cities Region was notably more affordable for housing and transportation and offered more diverse housing choices compared with similar metropolitan areas [17]. Those features may have fostered more convenient access to restaurants and small food stores. Second, the multidimensional class structure we identified by our data-driven approach is difficult to compare with class structure based on single features that other researchers have used. However, because of a lack of consistent association between individual neighborhood characteristics and specific food resource types [70], we elected to use our data-driven approach to characterize the neighborhood environment. Third, the marked undercount of food outlets in the D&B data may introduce bias [71]. Fourth, block groups were probably too small to reflect the service area of restaurants and food stores, especially in suburban areas. However, block group level data yield better estimates of the locations of food resources and households [72], compared with data from larger geographic units such as census tracts and zip codes. In addition, we could not obtain some retrospective built environment and sociodemographic data, such as traffic and crime, for the whole region, which have been suggested as relevant factors [73, 74]. W interpolated the sociodemographic factors at the block group level from the tract level for year 1993, the results of which may be inaccurate in rural areas and highly developed urban cores [75]. In addition, our models failed to converge when we included a change variable termed “the change in neighborhood type.” We expect to explicitly incorporate the change in neighborhood type to predict the change in neighborhood food availability when we have data for a greater number of observational years.

## Conclusion

We used cluster analysis to characterize food-related urban environments in the Twin Cities Region and examined the relationships between neighborhood type and relative availability of sit-down restaurants and supermarkets. We observed a complex and increasingly varied distribution of restaurants and food stores across six types of neighborhoods with distinctive built environment and sociodemographic characteristics, particularly for inner cities, during an 18-year time span. The composite index generated by cluster analysis and the associated food retailing landscaping provide an analytical tool to support public health policy in monitoring the neighborhoods that experienced great change in food availability. Our results echoed the national trend that the U.S. inner cities have undergone substantial changes in sociodemographic and built environment characteristics, which may subsequently or concurrently impact the types and distribution of restaurants therein. The great change in food outlet type in inner cities may have health implications for people who reside or work in such neighborhoods and who rely on such restaurants.

## Supplementary Information

**Additional file 1.** SIC codes used to identify food outlets.

**Additional file 2.** Robustness test of the optimal number of clusters.

**Additional file 3.** Neighborhood characteristics by neighborhood type in three observational years.

**Additional file 4.** Model results.

## Data Availability

The data that support the findings of this study are available from Carolina Population Center. Restrictions apply to availability because the data were licensed for the current study, and, therefore, not publicly available.

## Abbreviation

Twin Cities Region: Twin Cities Region of Minnesota
