# Supplementary material for: A method for estimating neighborhood characterization in studies of the association with availability of sit-down restaurants and supermarkets
Source: Int J Health Geogr. 2021 Mar 25;20:15. doi: 10.1186/s12942-020-00257-7 (PMC7995746; doi:10.1186/s12942-020-00257-7)
Supplement: Supplementary file 1 — Additional file 1. SIC codes used to identify food outlets. [file 12942_2020_257_MOESM1_ESM.docx]

Additional File 1: SIC codes used to identify food outlets

Table S1 Primary Standard Industrial Classification (SIC) codes from Dun & Bradstreet (D&B) used in the analysis in years 1993, 2001 and 2011, 8-digit codes shown below

| Food Resource Type | Description | D&B primary SIC code |
| --- | --- | --- |
| Sit-down Restaurants | Ethnic food restaurant  American restaurant  Cajun restaurant  Chinese restaurant  French restaurant  German restaurant  Greek restaurant  India/Pakistan restaurant  Italian restaurant  Japanese restaurant  Korean restaurant  Lebanese restaurant  Spanish restaurant  Thai restaurant  Vietnamese restaurant  Pakistani restaurant  Seafood restaurants: include sushi restaurants, oyster bars & seafood shacks  Steak house & BBQ restaurants  Chicken restaurants  Family-owned restaurant  Family-owned restaurants, chain  Family-owned restaurant, independent | 58120100  58120101  58120102  58120103  58120104  58120105  58120106  58120107  58120108  58120109  58120110  58120111  58120113  58120115  58120116  58120117  58120114  58120700  58120701  58120702  58120800  58120801  58120802  58129904  58120500  58120501  58120502 |
| Fast food Restaurants | Fast food restaurants and stands  Box lunch stand  Carry-out only (except pizza) restaurant  Chili stand  Coffee shop  Delicatessen (eating places)  Drive-in restaurant  Fast-food restaurant, chain  Fast-food restaurant, independent  Food bars  Pizzeria, Chain  Grills (eating places)  Hamburger stand  Hot dog stand  Sandwiches and submarines shop  Snack bar  Snack shop  Pizza restaurants  Pizzeria, chain  Pizzeria, independent | 58120300  58120301  58120302  58120303  58120304  58120305  58120306  58120307  58120308  58120309  58120601  58120310  58120311  58120312  58120313  58120314  58120315  58120600  58120601  58120602 |
| Supermarkets | Supermarkets  Supermarkets, chain  Supermarkets, independent  Supermarkets, greater than 100,000 square feet (hypermarket)  Supermarkets, 55,000-65,000 square feet (superstore)  Supermarket, 66,000-99,000 square feet | 54110100  54110101  54110102  54110103  54110104  54110105 |
| Grocery stores | Grocery store  Grocery store, nec  Frozen food and freezer plans, except meat  Country general stores  Grocery stores, chain  Grocery stores, independent | 54110000  54119900  54119903  53999903  54119904  54119905 |
| Convenience stores | Variety stores  Convenience stores  Convenience stores, chain  Convenience stores, independent  Gasoline service stations  Gasoline service stations, nec  Filling stations, gasoline | 53310000  54110200  54110201  54110202  55410000  55419900  55419901 |
