# Supplementary material for: A method for estimating neighborhood characterization in studies of the association with availability of sit-down restaurants and supermarkets
Source: Int J Health Geogr. 2021 Mar 25;20:15. doi: 10.1186/s12942-020-00257-7 (PMC7995746; doi:10.1186/s12942-020-00257-7)
Supplement: Supplementary file 2 — Additional file 2. Robustness test of the optimal number of clusters. [file 12942_2020_257_MOESM2_ESM.docx]

Additional File 2: Robustness test of the optimal number of clusters

Optimal number of clusters

We used three approaches to identify the goodness of the number of clusters we chose, the Gap Statistic Method, Average Silhouette Method, and Elbow Method. The Gap Statistic Method suggested six clusters were the optimal number to group our data. The Average Silhouette Method suggested seven clusters were optimal, and the Elbow Method suggested six clusters was the optimal number. We used the packages of *cluster*, *ggpubr*, *factoextra*, and *purr* in R x64 4.0.2,


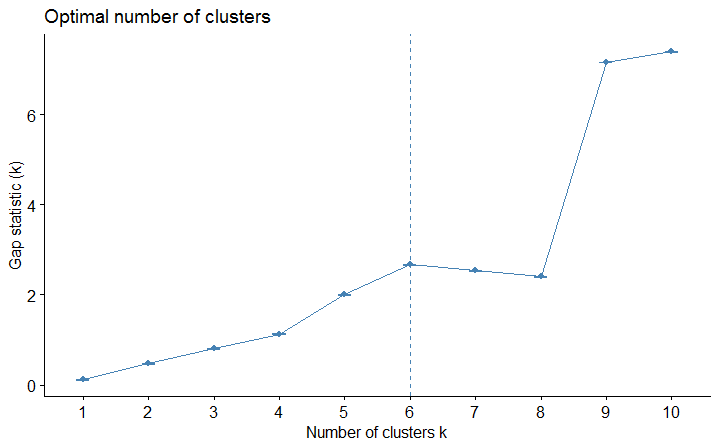


Figure S1 Gap Statistic Method to identify the optimal number of clusters


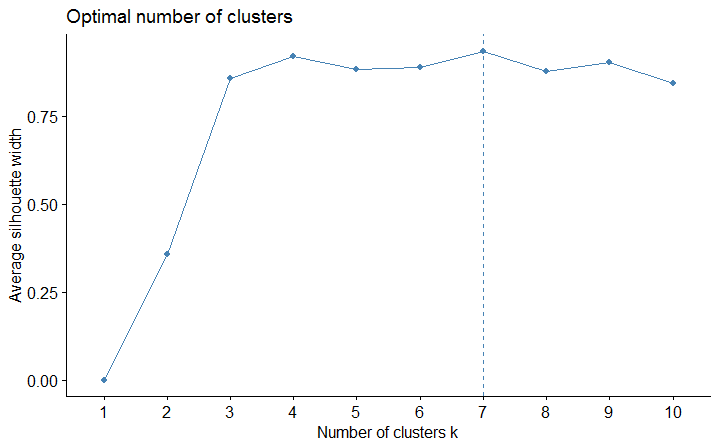


Figure S2 Average Silhouette Method to identify the optimal number of clusters


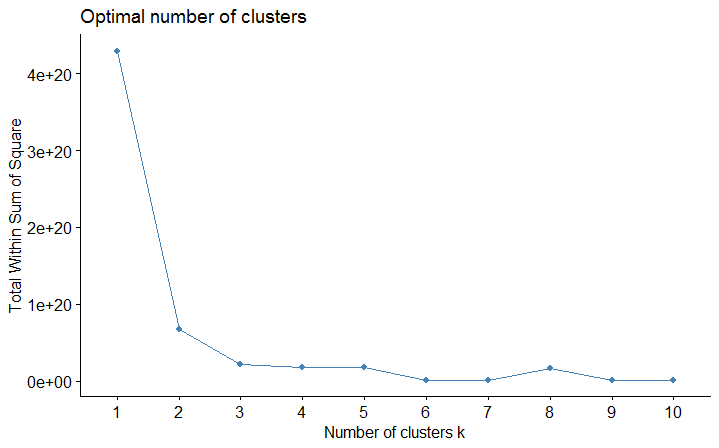


Figure S3 Elbow Method to identify the optimal number of clusters
