# Supplementary material for: A method for estimating neighborhood characterization in studies of the association with availability of sit-down restaurants and supermarkets
Source: Int J Health Geogr. 2021 Mar 25;20:15. doi: 10.1186/s12942-020-00257-7 (PMC7995746; doi:10.1186/s12942-020-00257-7)
Supplement: Supplementary file 3 — Additional file 3. Neighborhood characteristics by neighborhood type in three observational years. [file 12942_2020_257_MOESM3_ESM.docx]

Additional File 3: Neighborhood characteristics by neighborhood type in three observational years

Table S1. Specific neighborhood characteristics ^a^ by neighborhood type classified in 1993

|  | Urban core  (n=63) | Inner city  (n=103) | Urban  (n=335) | Aging suburb (n=579) | High-income suburb (n=331) | Suburban edge (n=672) |
| --- | --- | --- | --- | --- | --- | --- |
| **Built environment** |  |  |  |  |  |  |
| Residential population density,  1,000 person/km^2^ | 5.26±1.79 | 3.68±1.24 (1.62) | 2.45±1.03 (0.70) | 1.66±0.80 (0.11) | 0.87±0.48 | 0.43 (0.71)* |
| Employment population density,  1,000 person/km^2^ | 3.32±1.37 | 1.37±0.62 (0.74) | 1.41±0.57 | 0.84±0.39 (0.05) | 0.49±0.28 | 0.24 (0.40)* |
| Mix of land use, % ^b^ | 55.19±22.15 | 48.41±23.91 | 55.61±24.84 | 55.54±25.39 | 34.00 (49.00)* | 22.00 (39.00)* |
| % single-family housing ^c^ | 17.47 (28.74)* | 40.29±24.53 | 26.70 (38.54)* | 50.63±30.98 | 88.34 (41.90)* | 92.85 (21.79)* |
| **Sociodemographic** |  |  |  |  |  |  |
| % population aged under 14 ^d^ | 7.49±3.85^a^ | 28.09±7.51 | 17.16±4.51 | 20.08±4.18 | 20.27±3.42 | 28.13±2.98 |
| % population aged between 15 and 29 ^d^ | 50.76±14.21 | 25.11±4.82 | 26.84±7.02 | 22.64±3.88 | 19.32±4.03 | 22.56±3.61 |
| % population aged between 30 and 44 ^d^ | 22.41±7.10 | 23.63±3.31 | 29.28±4.22 | 23.69±2.75 | 25.63±3.11 | 30.83±3.40 |
| % population aged between 45 and 64 ^d^ | 10.13±4.85 | 12.44±2.83 | 15.01±3.18 | 17.93±3.42 | 24.48±3.49 | 14.60±3.08 |
| % population aged 65 or above ^d^ | 9.25±6.30 | 10.71±4.46 | 11.66±4.61 | 15.61±6.18 | 10.30±4.96 | 3.87±2.31 |
| Median household income, 1,000 $ ^e^ | 20.01±6.11 | 17.6±5.59 | 33.83±8.06 | 31.04±5.93 | 52.13±11.98 | 45.79±6.54 |
| % white race ^d^ | 85.00 (17.00)* | 44.21±16.98 | 93.00 (10.00)* | 95.00 (5.00)* | 97.00 (2.00)* | 97.00 (2.00)* |
| % black race ^d^ | 6.00 (9.00)* | 35.05±20.11 | 3.00 (5.00)* | 2.00 (3.00)* | 1.00 (1.00)* | 1.00 (1.00)* |
| % population with a college education or above ^d^ | 70.81±10.80 | 38.55±9.19 | 66.80±12.24 | 45.06±10.29 | 70.18±10.42 | 59.28±12.12 |

Note. *indicates that the values are skewed within a certain type of neighborhood and, therefore median (IQR) were used to summarize the characteristics. Otherwise, mean and standard error were used to summarize the characteristics.

^a^ Mean ± standard error (mean z-score) of neighborhood characteristics measured at the census block group level.

^b^ The mix of land use was measured by 3-tier land use entropy (denominator set to the static 3 land use types in the census block group), which used three land use categories (residential, employment and retail) to calculate mix of land use in the census block group.

^c^ Percent of single-family housing relative to total single-family and multi-family housings.

^d^ The denominators of percent of population aged under 14, aged between 15 and 29, aged between 30 and 44, aged between 45 and 64, aged 65 or above, population with a college education or above, white race and black race were total population in the census block group.

^e^ The median household income in 1993 and 2001 were adjusted for inflation to compare with that in 2011.

Table S2. Specific neighborhood characteristics ^a^ by neighborhood type classified in 2001

|  | Urban core  (n=63) | Inner city  (n=103) | Urban  (n=335) | Aging suburb (n=579) | High-income suburb (n=331) | Suburban edge (n=672) |
| --- | --- | --- | --- | --- | --- | --- |
| **Built environment** |  |  |  |  |  |  |
| Residential population density, 1,000 person/km^2^ | 4.97±1.83 | 4.00±1.70 | 2.40±1.43 | 1.68±1.01 | 1.04±0.59 | 0.67 (0.73)* |
| Employment population density, 1,000 person/km^2^ | 3.14±1.46 | 1.68±0.90 | 1.39±0.87 | 0.85±0.47 | 0.57±0.35 | 0.35 (0.42)* |
| Mix of land use, % ^b^ | 54.36±30.00 | 60.68±26.20 | 52.57±27.85 | 59.78±27.46 | 42.84±29.01 | 33.11 (47.40)* |
| % single-family housing ^c^ | 20.25 ( 8.87)* | 37.80±24.62 | 28.98 (34.89)* | 54.80±31.31 | 90.99 (39.73)* | 95.91 (17.36)* |
| **Sociodemographic** |  |  |  |  |  |  |
| % population aged under 14 ^d^ | 7.65 (7.27)* | 25.70±9.75 | 16.86±6.08 | 20.63±5.75 | 19.21±4.82 | 25.50±4.70 |
| % population aged between 15 and 29 ^d^ | 42.07±13.47 | 26.89±6.32 | 24.88±9.33 | 20.28±4.94 | 17.55±5.51 | 18.07±4.29 |
| % population aged between 30 and 44 ^d^ | 24.65 (11.27)* | 24.73±5.21 | 26.56±4.83 | 24.59±3.88 | 23.64±4.04 | 28.40±4.50 |
| % population aged between 45 and 64 ^d^ | 15.61±5.96 | 15.23±3.78 | 20.30±4.67 | 20.37±4.35 | 25.17±4.60 | 22.00±5.33 |
| % population aged 65 or above ^d^ | 5.21 (9.79)* | 6.09 (6.90)* | 11.39±6.99 | 13.80±7.37 | 14.41±8.02 | 6.03±4.10 |
| Median household income, 1,000 $ ^e^ | 24.08±9.09 | 24.34±7.82 | 37.20±10.85 | 36.09±9.52 | 48.86±14.92 | 53.57±13.09 |
| % white race ^d^ | 79.94 (31.97)* | 44.12±23.04 | 86.63±16.57 | 86.67 (16.86)* | 92.49 (7.29)* | 93.52 (5.95)* |
| % black race ^d^ | 9.29±19.35 | 29.31±16.90 | 4.52 (7.98)* | 3.65 (6.85)* | 2.01 (2.75)* | 1.27 (2.20)* |
| % population with a college education or above ^d^ | 73.20±15.74 | 48.20±16.66 | 74.34 (19.11)* | 57.59±13.2 | 73.74±10.39 | 69.02±11.79 |

*indicates that the values are skewed within a certain type of neighborhood and therefore median (IQR) were used to summarize the characteristics. Otherwise, mean and standard error were used to summarize the characteristics.

^a^ Mean ± standard error (mean z-score) of neighborhood characteristics measured at the census block group level.

^b^ The mix of land use was measured by 3-tier land use entropy (denominator set to the static 3 land use types in the census block group), which used three land use categories (residential, employment and retail) to calculate mix of land use in the census block group.

^c^ Percent of single-family housing relative to total single-family and multi-family housings.

^d^ The denominators of percent of population aged under 14, aged between 15 and 29, aged between 30 and 44, aged between 45 and 64, aged 65 or above, population with a college education or above, white race and black race were total population in the census block group.

^e^ The median household income in 1993 and 2001 were adjusted for inflation to compare with that in 2011.

Table S3. Specific neighborhood characteristics ^a^ by neighborhood type classified in 2011

|  | Urban core  (n=63) | Inner city  (n=103) | Urban  (n=335) | Aging suburb  (n=579) | High-income suburb (n=331) | Suburban edge (n=672) |
| --- | --- | --- | --- | --- | --- | --- |
| **Built environment** |  |  |  |  |  |  |
| Residential population density, 1,000 person/km^2^ | 5.92±1.87 | 3.58±1.56 | 2.47±1.11 | 1.69±0.85 | 0.89±0.46 | 0.79 (0.80) * |
| Employment population density, 1,000 person/km^2^ | 3.82±1.51 | 1.53±0.91 | 1.45±0.68 | 0.84±0.40 | 0.47±0.25 | 0.43 (0.45) * |
| Mix of land use, % ^b^ | 56.70±27.01 | 61.49±23.73 | 61.64±23.38 | 70.00 (35.00)* | 47.75±28.58 | 37.00 (57.00) * |
| % single-family housing ^c^ | 0.00 (52.81)* | 52.94±38.18 | 77.04 (69.26)* | 91.45 (30.79)* | 96.90 (25.54) * | 99.86 (9.59) * |
| **Sociodemographic** |  |  |  |  |  |  |
| % population aged under 14 ^d^ | 7.00 (9.00)* | 26.66±8.18 | 17.08±5.36 | 19.19±5.37 (-0.34) | 18.81±3.82 | 22.50±4.22 |
| % population aged between 15 and 29 ^d^ | 45.00 (21.00) * | 27.06±6.43 | 24.54±8.05 | 21.40±4.76 (-0.15) | 16.43±4.13 | 19.43±4.11 |
| % population aged between 30 and 44 ^d^ | 20.13±8.66 | 20.26±4.35 | 24.48±4.50 | 21.47±4.06 (-0.73) | 18.78±3.17 | 22.89±3.72 |
| % population aged between 45 and 64 ^d^ | 14.65±5.85 | 19.00±5.59 | 24.26±5.06 | 24.17±4.02 (0.20) | 30.41±4.53 | 27.65±4.66 |
| % population aged 65 or above ^d^ | 5.00 (2.00)* | 6.00 (5.00)* | 9.68±3.86 | 13.75±6.66 (0.87) | 15.49±5.44 | 7.48±3.28 |
| Median household income, 1,000 $ ^e^ | 19.24±7.84 | 18.37±6.39 | 36.22±11.36 | 30.57±6.56 (-0.60) | 49.86±13.54 | 48.95±11.34 |
| % white race ^d^ | 75.83±14.63 | 34.77±17.75 | 84.00 (19.00) * | 83.00 (22.00) * | 92.00 (9.00)* | 89.00 (10.00) * |
| % black race ^d^ | 10.00 (12.00)* | 38.50±16.65 | 6.00 (10.00) * | 6.00(9.00) * | 2.00 (4.00) * | 2.00 (4.00) * |
| % population with a college education or above ^d^ | 74.52±14.30 | 44.21±12.21 | 75.30±13.17 | 58.54±10.90) | 77.69±9.91 | 71.22±10.38 |

*indicates that the values are skewed within a certain type of neighborhood and therefore median (IQR) were used to summarize the characteristics. Otherwise, mean and standard error were used to summarize the characteristics.

^a^ Mean ± standard error (mean z-score) of neighborhood characteristics measured at the census block group level.

^b^ The mix of land use was measured by 3-tier land use entropy (denominator set to the static 3 land use types in the census block group), which used three land use categories (residential, employment and retail) to calculate mix of land use in the census block group.

^c^ Percent of single-family housing relative to total single-family and multi-family housings.

^d^ The denominators of percent of population aged under 14, aged between 15 and 29, aged between 30 and 44, aged between 45 and 64, aged 65 or above, population with a college education or above, white race and black race were total population in the census block group.

^e^ The median household income in 1993 and 2001 were adjusted for inflation to compare with that in 2011.

Table S4 Average number of sit-down restaurants by neighborhood type

|  | Urban core | Inner city | Urban | Aging suburb | High-income suburb | Suburban edge |
| --- | --- | --- | --- | --- | --- | --- |
| 1993 | 1.0 | 0.4 | 0.6 | 0.5 | 0.3 | 0.2 |
| 2001 | 1.3 | 0.6 | 0.8 | 0.6 | 0.6 | 0.4 |
| 2011 | 2.0 | 1.2 | 1.2 | 0.9 | 0.9 | 0.9 |
