# Supplementary material for: A method for estimating neighborhood characterization in studies of the association with availability of sit-down restaurants and supermarkets
Source: Int J Health Geogr. 2021 Mar 25;20:15. doi: 10.1186/s12942-020-00257-7 (PMC7995746; doi:10.1186/s12942-020-00257-7)
Supplement: Supplementary file 4 — Additional file 4. Model results. [file 12942_2020_257_MOESM4_ESM.docx]

Additional File 4: Model results

Table S1. Predicted multivariable-adjusted model coefficients of associations among the percent of sit-down restaurants relative to the total of sit-down restaurants and fast food restaurants, neighborhood type in 1993, interaction of the latter with time elapsed, and time elapsed from years 1993, 2001 and 2011: Twin Cities Region of Minnesota

| Predictors | Block group model | | Tract model | | Place model | |
| --- | --- | --- | --- | --- | --- | --- |
|  | b (95% CI) | P value | b (95% CI) | P value | b (95% CI) | P value |
| Neighborhood type in 1993 ^a^ |  |  |  |  |  |  |
| Urban core | **24.78 (15.01, 34.54)** | **0.000** | **25.52 (14.96, 36.09)** | **0.000** | 0.09 (-4.41, 4.59) | 0.970 |
| Inner city (Ref) | --- | --- | --- | --- | --- | --- |
| Urban | -0.86 (-7.73, 6.01) | 0.806 | **9.55 (2.11, 16.99)** | **0.012** | -3.13 (-6.29, 0.04) | 0.053 |
| Aging suburb | -2.60 (-9.12, 3.92) | 0.434 | -1.13 (-8.20, 5.93) | 0.753 | **-6.73 (-9.74, -3.73)** | **0.000** |
| High-income suburb | -4.56 (-11.44, 2.32) | 0.194 | -2.79 (-10.24, 4.66) | 0.463 | **-10.04 (-13.21, -6.87)** | **0.000** |
| Suburban edge | **-7.87 (-14.32, -1.42)** | **0.017** | **-9.32 (-16.31, -2.34)** | **0.009** | **-17.48 (-20.45, -14.50)** | **0.000** |
|  |  |  |  |  |  |  |
| Neighborhood type in 1993: time elapsed ^b^ |  |  |  |  |  |  |
| Urban core | **-1.15 (-1.84, -0.45)** | **0.001** | -1.50 (-2.25, -0.76) | **0.000** | -0.01 (-0.31, 0.29) | 0.956 |
| Inner city (Ref) | --- | --- | --- | --- | --- | --- |
| Urban | **-0.53(-1.02, -0.04)** | **0.032** | **-1.18 (-1.70, -0.65)** | **0.000** | 0.01 (-0.20, 0.23) | 0.901 |
| Aging suburb | **-0.58 (-1.05, -0.12)** | **0.013** | **-1.03 (-1.52, -0.53)** | **0.000** | -0.09 (-0.30, 0.11) | 0.360 |
| High-income suburb | -0.33 (-0.82, 0.16) | 0.182 | -0.40 (-0.92, 0.12) | 0.132 | 0.07 (-0.15, 0.28) | 0.538 |
| Suburban edge | -0.38 (-0.84, 0.07) | 0.098 | **-0.56 (-1.05, -0.07)** | **0.025** | **0.20 (0.00, 0.40)** | **0.050** |
|  |  |  |  |  |  |  |
| Time elapsed ^c^ | **0.78 (0.35, 1.21)** | **0.000** | **1.05 (0.60, 1.52)** | **0.000** | -0.12 (-0.31, 0.07) | 0.203 |
|  |  |  |  |  |  |  |
| Covariates |  |  |  |  |  |  |
| Change in employment population density, 1,000 person/km^2^ | **2.82 (0.35, 5.28)** | **0.025** | **0.35 (-5.70, 5.00)** | **0.900** | **0.97 (-0.00, 0.00)** | 0.082 |
| Change in income, 1,000 US dollar | 0.08 (-0.03, 0.18) | 0.147 | -0.06 (-0.26, 0.15) | 0.597 | -0.04 (-0.08, 0.01) | 0.099 |
| Change in percent of white | 0.03 (-0.06 0.11) | 0.564 | 0.02 (-0.11, 0.15) | 0.768 | -0.03 (-0.07, 0.01) | 0.089 |
| Change in percent of single family housing | 0.02 (-0.03, 0.07) | 0.420 | 0.05 (-0.01, 0.10) | 0.099 | 0.02 (-0.00, 0.04) | 0.082 |
| Total of sit-down restaurants and fast food restaurants, count | **2.98 (2.68, 3.26)** | **0.000** | **0.96 (0.65, 1.26)** | **0.000** | **0.19 (0.06, 0.33)** | **0.004** |
|  |  |  |  |  |  |  |
| Constant | **17.94 (11.93, 23.95)** | **0.000** | **33.95 (27.44, 40.46)** | **0.000** | **54.80 (52.03, 57.58)** | **0.000** |

Abbreviations: b: model effect; CI: confidential interval. **Bold** font indicates significant association (P <.05). N=6,249.

^a^ The coefficient of neighborhood type in 1993 shows if other types of neighborhoods had a greater percent of sit-down restaurants than the reference neighborhood type (inner city) in 1993.

^b^ Time elapsed in 1993, 2001, and 2011 is defined as 0, 8, and 18, respectively. The coefficient of the interaction term between neighborhood type in 1993 and the time elapsed shows if other types of neighborhoods experienced a greater increase in the percent of sit-down restaurants than the reference neighborhood type (inner city).

^c^ The coefficient of time elapsed refers to the effect of time on the reference neighborhood type (inner city). The coefficient of time elapsed shows if the reference neighborhood type experienced a significant change in the percent of sit-down restaurants between 1993 and 2011.

Table S2. Predicted multivariable-adjusted model coefficients of associations among the percent of supermarkets relative to the total of supermarkets, grocery stores and convenience stores, neighborhood type in 1993, interaction of the latter with time elapsed, and time elapsed from years 1993, 2001 and 2011: Twin Cities Region of Minnesota

| Predictors | Block group model | | Tract model | | Place model | |
| --- | --- | --- | --- | --- | --- | --- |
|  | b (95% CI) | P value | b (95% CI) | P value | b (95% CI) | P value |
| Neighborhood type in 1993 ^a^ |  |  |  |  |  |  |
| Urban core | 1.87 (-2.50, 6.24) | 0.401 | 0.69 (-5.19, 6.58) | 0.817 | -0.38 (-2.16, 1.40) | 0.673 |
| Inner city (Ref) | --- | --- | --- | --- | --- | --- |
| Urban | 0.46 (-2.67, 3.59) | 0.773 | 0.28 (-3.88, 4.44) | 0.895 | -0.01 (-1.28, 1.27) | 0.991 |
| Aging suburb | 1.69 (-1.39, 4.77) | 0.282 | 2.08 (-2.06, 6.22) | 0.324 | 0.46 (-0.80, 1.71) | 0.473 |
| High-income suburb | 1.30 (-2.07, 4.66) | 0.450 | -0.08 (-4.65, 4.50) | 0.974 | 1.14 (-0.23, 2.51) | 0.104 |
| Suburban edge | 0.19 (-3.08, 3.46) | 0.909 | -1.35 (-5.83, 3.12) | 0.554 | -0.14 (-1.47, 1.19) | 0.833 |
|  |  |  |  |  |  |  |
| Neighborhood type in 1993: time elapsed ^b^ |  |  |  |  |  |  |
| Urban core | 0.02 (-0.29, 0.33) | 0.910 | **0.43 (0.04, 0.83)** | **0.032** | -0.01 (-0.14, 0.12) | 0.895 |
| Inner city (Ref) | --- | --- | --- | --- | --- | --- |
| Urban | -0.07 (-0.29, 0.15) | 0.546 | 0.05 (-0.23, 0.33) | 0.708 | 0.02 (-0.07, 0.11) | 0.615 |
| Aging suburb | 0.00 (-0.21, 0.21) | 0.990 | 0.01 (-0.25, 0.28) | 0.931 | -0.02 (-0.11, 0.06) | 0.595 |
| High-income suburb | -0.15 (-0.37, 0.07) | 0.182 | 0.02 (-0.26, 0.29) | 0.915 | -0.03 (-0.12, 0.06) | 0.481 |
| Suburban edge | -0.00 (-0.21, 0.20) | 0.988 | 0.21 (-0.05, 0.47) | 0.118 | **0.09 (0.01, 0.18)** | **0.033** |
|  |  |  |  |  |  |  |
| Time elapsed ^c^ | **0.24 (0.05, 0.43)** | **0.014** | 0.18 (-0.07, 0.42) | 0.157 | **0.25 (0.17, 0.33)** | **0.000** |
|  |  |  |  |  |  |  |
| Covariates |  |  |  |  |  |  |
| Change in residential population density, 1,000 person/km^2^ | 0.04 (-0.43, 0.51) | 0.869 | 0.23 (-0.52, 0.98) | 0.547 | -0.07 (-0.26, 0.13) | 0.500 |
| Change in income, 1,000 US dollar | -0.04 (-0.08, 0.01) | 0.101 | -0.04 (-0.15, 0.07) | 0.494 | **-0.05 (-0.07, -0.03)** | **0.000** |
| Change in percent of white | 0.02 (-0.02, 0.06) | 0.356 | -0.03 (-0.10, 0.05) | 0.495 | -0.00 (-0.02, 0.01) | 0.617 |
| Change in percent of single-family housing | **-0.03 (-0.05, -0.01)** | **0.016** | -0.02 (-0.05, 0.01) | 0.184 | 0.01 (0.01, 0.02) | **0.002** |
| Total of supermarkets, grocery stores and convenience stores, count | **1.83 (1.51, 2.16)** | **0.000** | 0.40 (-0.09, 0.88) | 0.108 | -0.04 (-0.17, 0.09) | 0.535 |
|  |  |  |  |  |  |  |
| Constant | -0.83 (-4.08, 2.42) | 0.616 | 3.39 | 0.140 | **3.96 (2.64, 5.29)** | **0.000** |

Abbreviations: b: model effect; CI: confidential interval. **Bold** font indicates significant association (P <.05). N=6,249.

^a^ The coefficient of neighborhood type in 1993 shows if other types of neighborhoods had a greater percent of supermarkets than the reference neighborhood type (inner city) in 1993.

^b^ Time elapsed in 1993, 2001, and 2011 is defined as 0, 8, and 18, respectively. The coefficient of the interaction term between neighborhood type in 1993 and the time elapsed shows if other types of neighborhoods experienced a greater increase in the percent of supermarkets than the reference neighborhood type (inner city).

^c^ The coefficient of time elapsed refers to the effect of time on the reference neighborhood type (inner city). The coefficient of time elapsed shows if the reference neighborhood type experienced a significant change in the percent of supermarkets between 1993 and 2011.
